# Work-hour Restrictions: Respecting Individual Wishes

**DOI:** 10.31662/jmaj.2025-0003

**Published:** 2025-03-07

**Authors:** Shigeki Matsubara

**Affiliations:** 1 Department of Obstetrics and Gynecology, Jichi Medical University, Tochigi, Japan; 2 Department of Obstetrics and Gynecology, Koga Red Cross Hospital, Koga, Japan; 3 Medical Examination Center, Ibaraki Western Medical Center, Chikusei, Japan

**Keywords:** education, skill, training, resident, work hour

## Dear Editors,

Doctor Nagasaki’s opinion ^(1)^ is highly significant. He questioned how work-hour restrictions might affect residents’ future performance. Will current residents have satisfactory practice and research skills in 20 years? My conclusion is: nobody knows. However, I wish to highlight an “invisible” issue suggesting a clue: the mindset of residents shaped by living their residency under these restrictions and the atmosphere surrounding them.

Nobody knows? Factors supporting the view that “residents’ ability will not decrease” include: i) technological advancement may make time-efficient training possible ^(2)^, ii) increased support from non-doctor staff, and iii) private time boosting energy for work and research. Factors suggesting “their ability will decrease” are: i) rising demands for advanced skills (e.g., robotic and endoscopic surgery) and ii) reduced time for acquiring such skills. As these influences counterbalance, the outcome remains uncertain.

I do not wish to claim that “the harder, the better” shaped me. During my residency, the older generation often said the same. In hindsight, nostalgia for the past might lead one to justify such views. However, we, seasoned doctors, must never forget that overwork causes burnout and even death (*karo-shi*) ^(3)^. Bearing this in mind, we must consider the mindset and atmosphere that work-hour restrictions may foster.

First, some residents strongly aspire to become “world-class.” The mindset of “leaving the hospital early” may deprive them of opportunities to voluntarily engage in surgery or research. Second, overemphasis on “no work, no pay” and “with work, with pay” risks eroding the volunteer spirit of younger generations, reducing hospital time to mere monetary terms. Third, if 90% consider “leaving immediately” the norm, the remaining 10% may feel that staying late is inherently wrong.

I fundamentally agree with work-hour restrictions; however, I believe personal “wishes” and “individuality” should also be respected. Why do we choose to become doctors? For some, it may be the higher income per hour, a valid reason I do not criticize. Yet, for many, it stems from a strong desire to help patients and to grow as “good” human beings through shared time with them. Laborious efforts serve not only patients but also our own personal growth.

I hope ambitious residents eager for “harder” work and driven by voluntary spirit are respected. Let them follow their wishes, importantly, provided senior doctors safeguard their mental and physical health. “Heterogeneity” is fundamental to both humanity and medical society. This principle applies to work-hour restrictions as well.

## Article Information

### Conflicts of Interest

None

### Author Contributions

S. Matsubara.

Identification of the significance. Manuscript writing.

### Approval by Institutional Review Board (IRB)

Not applicable.

### Patient Anonymity

Not applicable.

### Informed Consent

Not applicable.
